# A gender-based investigation into the required English language policies in Saudi higher education institutions

**DOI:** 10.1371/journal.pone.0274119

**Published:** 2022-09-09

**Authors:** Suliman Mohammed Nasser Alnasser

**Affiliations:** Department of English Language and Literature, College of Arts, King Saud University, Riyadh, Saudi Arabia; North-West University Potchefstroom Campus: North-West University, SOUTH AFRICA

## Abstract

The Saudi Arabian higher education system is yet to address specific issues concerning language planning and policy. Policies concerning English language use in Saudi academia remain ungoverned, thus leading to implicit personal policies (i.e., self-directed policies). Such freedom in language use may generate concerns among stakeholders, especially if differences emerge between men and women in the same workplace. Current literature does not offer insights into how English is used by each gender in academic settings or outside the classroom in Saudi Arabia; this study seeks to address this gap. A six-item online survey was distributed among higher education English language departments in Saudi Arabia. The questionnaire was completed by 210 faculty members (67 men and 143 women). Responses from men and women were compared using the Mann-Whitney test. The main findings include significant differences in language use between the two genders, suggesting different levels of commitment to English use. These findings imply that future English language policies may need to incorporate divergent views between the two genders. The recommendations include the need for micro-level policies and the involvement of both genders in designing policies to ensure their successful application.

## Introduction

In a multilingual country, language governance is often needed for varied purposes, including creating an attractive economic context and reducing possible communication conflicts within a community [1, 2]. The governance of languages in English as a foreign language (EFL) context can be achieved through English language policies (ELPs), which can play a prominent role in the management of language use and facilitate the achievement of goals set by a government or educational institution [3]. In Saudi Arabia, English language departments exist in most higher education institutions; they offer English education at all graduate and postgraduate levels [4]. These departments employ faculty members from within the country, the wider Arab world, and other non-Arabic-speaking countries. This cultural diversity among faculty might require language governance as current approaches to English use are arbitrary and somewhat complicated [5].

It is commonly accepted that the dominance of the English language around the globe influences its status in different EFL contexts. For instance, the presence of Western culture in the Gulf has been strongly impacted by reliance on expatriate experts for infrastructure development. Currently, English serves as a *lingua franca* for contact with expatriates working in the public and private sectors. Additionally, English has invaded local communities in magazines, the Internet, and television programmes, creating further exposure to the language. It is gradually invading the social landscape in streets, malls, and public and private sector facilities, leading to the incidental acquisition of English and the implicit and urgent need to develop language skills. Consequently, the younger generation has been influenced in a way that makes them uncertain about the importance of their L1; they may believe that mastering English is equally important as—or even more important than—learning Arabic [6–8]. In such a context, language planning and policies can play a distinctive role in managing how languages function within a community to achieve predefined objectives.

In recent years, ELPs in Saudi Arabia have gained increasing attention [5, 9], and a survey of the literature suggests that current ELPs are being practised informally, implicitly (without written guidelines), and freely [5]. In fact, it was found that current policies are being implemented according to personal preferences (i.e., self-directed ELPs) in that they are practised with no governance whatsoever. Such freedom in ELPs creates differences among individuals in a community regarding what language (L1 or L2) should be used in different communicative encounters and situations. To the best of my knowledge, the literature has paid scant attention to English use by faculty members in Saudi English departments and outside the domain of the classroom. Moreover, differences in these self-directed ELPs between male and female faculty have not been investigated, and exploring the level of agreement or disagreement between them can provide insights into how unified policies may be designed. Additionally, the findings may help appraise the urgency of designing ELPs at the micro level. Thus, the current study addresses the following research questions:

1. Are there gender differences among faculty members who practise self-directed ELPs in Saudi English departments?
2. If so, how can these differences be addressed in designing effective ELPs?

### General background on language planning and policy

According to Deumert [10 p. 384], Einar Haugen introduced the concept of ‘language planning’ half a century ago, which describes ‘all conscious efforts that aim at changing the linguistic behaviour of a speech community’. Deumert [10] further discussed that such a process may be as large as introducing a new language or as small as making word-level changes. Therefore, language planning is an administrative and political process aimed at resolving language-related issues within a community [11–13]. Similarly, Tauli [14] holds that language planning involves the ‘activity of regulating and improving existing languages or creating new common regional, national, or international languages’. In contrast, language policy maintains a more overarching nature. In the words of Deumert [10 p. 384], it refers to ‘the more general linguistic, political, and social goals underlying the actual language planning process’, while Kaplan and Baldauf [15] see it as the ‘body of ideas, laws, regulations, rules, and practices intended to achieve the planned language change in a society, group, or system’. The definitions provided here are indicative of the significance of language policy and planning for multilingual communities; they demonstrate how language use can be governed to facilitate improved outcomes.

Johnson [16] and Schiffman [17] classified language policies. According to them, these policies can be classified according to their degree of formality (implicit or explicit) and genesis—that is, whether they emerge from higher authorities and are passed down (macro-level policies), or vice versa (micro-level policies). Here, a relationship exists between these two classifications: namely, implicit (explicit) policies tend to exhibit a lower (higher) degree of formality. By their very nature, implicit policies might exist in a community and be widely practised by its members without their awareness.

### English in higher education: The Gulf region

Close observations of the Gulf region reveal that English is becoming increasingly dominant every year. Policymakers are intending to advocate education policies that promote even greater language use. Higher education reforms in the Gulf region are underway, centred on the assumption that English use is an integral part of the reforms as a language for instruction. Arguably, this is an ‘efficient shortcut’ for achieving educational reforms [18]. The recent emphasis on English in higher education institutions reflects the acceptance of the belief that English can precipitate positive educational changes by facilitating access to the knowledge produced by international scholars, enabling students to keep up with the developments in various disciplines [2, 19]. These institutions seemingly have a ‘hunger for English’ [20, p. 170], which is a growing phenomenon worldwide [21, 22], particularly as a perceived means of attracting international students [23].

The adoption of English as a medium of instruction in the Gulf region is an expanding practice among several universities. Badry and Willoughby [18] explored the views of officials and academics in these universities about this practice. Their interviews justified the reliance on English as the language of research, technology, and science. Furthermore, graduating students with proficient English language skills reportedly exhibited improved chances of successful employment. English skills can help graduates perform better and excel in their future studies and careers [24]. This view is shared by other researchers [19, 25, 26], who explain that English is becoming the vehicle of lifestyles and cultures in different communities. Moreover, this may foster the standardisation of ‘Western measures of skill, technology, innovation, and productivity in ways that are quickly recalibrating regional economic and political relationships’ [2]. However, setting priorities for English in education can diminish the role of the first language [27], and students may feel pressured to learn English and other subjects simultaneously [18, 28]. Here, Benson [29] and Trudell [30] concluded that learning in higher education might be of higher quality when the first language is employed as the language of instruction.

### Language policies in education

In multilingual contexts, issues concerning, for example, language use in the workplace may emerge, which require the intervention of decision makers to regulate the usage of language by enforcing certain language policies [20, 31]. In education, languages are used for communication, instruction, and other purposes. Accordingly, language policies in education have emerged prominently in multilingual contexts. Arguably, a country can positively influence its economy by establishing one language for education [19]. Some non-English-speaking countries have emphasised the role of their national language as a sign of unity while recognising the importance of English to facilitate local and international communication [32]. The usage of English is controversial in some countries, such as Somalia [33], South Asian countries [1, 20, 24], and the Philippines [31]. Tickoo [20] posited that educationalists’ beliefs have been influenced by Westernisation, which influences them to perceive a superior role for English in education. In Sweden, macro language policies have been enforced to preserve Swedish and limit the presence of English in the educational system [28, 34]. Universities are concerned both with preserving heritage and advancing knowledge [3], and the coexistence of the first language and English is often accepted to facilitate the achievement of overlapping goals [35].

Policymakers are often committed to internationalising and globalising their educational systems. This requires intervention at different levels of the educational system, including research and language governance [21, 36]. Arguably, higher education institutions play a prominent role in preserving societal values and promoting knowledge advancement to promote global competitiveness; this is accomplished through various practices, teachers, students, ideas, and knowledge. In fact, several international universities have enforced language policies advocating English to become the language of research, instruction, and communication—with the purpose of internationalising their educational setting and improving their ranking among competing universities [28]. Liddicoat and Kirkpatrick [30, p. 26] stated that the dominance of English worldwide is attributed to the ‘increasing globalised role of English and an ideological positioning of English as the language of modernisation and economic opportunity, supported by the neoliberal agenda of education for economic utility’.

In their study, Bolton and Kuteeva [37] interviewed 4524 students and 668 academics in different EFL contexts regarding their perceptions of adopting English as a language of instruction across different disciplines. They concluded that instruction in English is preferred in most, but not all, disciplines. Furthermore, Eno et al. [33, p. 113] explored the views of educational policymakers and academics on the use of English solely for instruction; their main conclusion was that English is preferred because it offers ‘opportunities for academic studies and professional advancement in the future’.

Although adopting English for instruction seems tempting for many policymakers, arguably, this can pressure EFL/ESL students and increase inequality among learners [38–41]. For example, in such contexts, learners might come from different educational backgrounds and have different levels of language proficiency. As a result, they are required to simultaneously improve their English language skills as well as master the knowledge in their chosen subject, which can be problematic. Further, several concerns have been raised concerning the validity and reliability of student assessment; it may not be ethical to test students’ knowledge in a language in which they might encounter difficulty in expressing their thoughts [39, 42, 43]. Such concerns have been expressed for decades but have remain overlooked [44]. Therefore, policymakers may not have exerted sufficient efforts in accomodating the learning context to students [42]. These concerns have led some EFL instructors to rely on L1 in their teaching in an attempt to ensure that their learners gain adequate knowledge. Students may suffer owing to linguistic deficiencies, and to ensure equal learning opportunities, some instructors tend to use ‘very little’ English [24].

### The Saudi EFL context: Language planning and policies

In the Saudi context, Arabic has been the official language for centuries. Arabs have been proud of Arabic because it is the language of their ancestors and, of course, the language of the Holy Quran. Some researchers see it as the holy language of Muslims around the globe [9, 45, 46], which reflects how sacred Arabic is to Muslims and explains why its use is being advocated in their communities [46]. Nonetheless, in recent years, the spread and acceptance of English by Saudi institutions and communities has accorded it a high status; this trend is imposed by the internationalisation of commerce, media, and education.

English is employed as a lingua franca in most official and non-official communication with non-Arabic speakers. For example, some official letters include Arabic and English texts in the letterhead section. Until recently, the Saudi Ministry of Education had only allowed English to be taught in addition to Arabic. English was taught as early as the fifth grade at the elementary level, and in early 2021, a decree was signed by the minister, deciding to teach English as early as the first elementary level (for children aged 6). Regarding higher education, Saudi students were offered innumerable opportunities to study abroad in English-speaking Western and European countries, which clearly established a need to master English to succeed academically. Such implicit enthusiasm for English has accorded it a high status, leading many people to accept it as a necessity for living a better life, in that it can facilitate receiving better service in restaurants and other private companies and help obtain better job opportunities.

The area of ELPs has attracted Saudi research in recent years [4, 9, 47, 48]. Language policies concerning English in the Saudi higher education system are found to be rather implicit in nature. The legislative document for higher education which has been approved by the Saudi Royal Court clearly states that Arabic is the language of instruction, though making exceptions for specific programmes is possible. Accordingly, several Bachelor’s, Master’s, and doctoral programmes in fields such as medicine, administration, pharmacy, business, and law, are being taught in English. A recent study by Almoaily and Alnasser [5] concluded that no clear policies exist to govern English use in the Saudi higher education system. Several Saudi academics in Saudi universities obtained their degrees from Western universities, which has influenced their use of English in the workplace [4]; they tend to use English to communicate with other faculty and students. With the absence of ELPs governing language use, the assumptions and gender differences behind the use of English remain ambiguous.

### Gender differences and language planning and policies

From a general perspective, the literature provides extensive studies examining the language differences between men and women in different communities. Significant differences exist between the two genders in terms of verbal performance, cognition [49], and attitudes towards languages [50]. Regarding learning, significant differences between the two genders were found in relation to the use of learning strategies [51, 52], motivation to become multilanguage speakers [53], and beliefs about learning strategies [54]. Moreover, research suggests that men and women tend to use different linguistic interactive patterns [55] and show different degrees of speech [56]; for example, men tend to be more assertive in discussions [56, 57]. Conversely, Bernat and Lloyd [58] did not find significant differences between male and female students regarding their beliefs and preferences regarding language learning.

Language policies in an educational context (such as English language departments) will be implemented by both men and women. Since the literature has shown that ELPs in the Saudi higher educational context do not exist formally and explicitly [5], male and female instructors are likely to practice their own self-directed policies, which may embody gender differences. One study identified gender differences in Saudi English departments [4], which is perhaps unsurprising given that women have been segregated from men in the academic workplace for decades. The study [4] demonstrated that Saudi women have a tendency to be more assertive in their views than men. Here, the segregation may arguably lead to acquiring distinctive linguistic features from one another. Such differences could potentially be alleviated if the segregation was ended, promoting a greater degree of direct contact between the two genders [59]. Investigating such differences can provide insights into how men and women practise their own ELPs and, therefore, inform policymakers about the type of policies that each gender expects.

## Method

The study design was approved by the appropriate ethics review board at King Saud University. The participants indicated their consent to participate in the study by agreeing to complete the online survey. A quantitative approach to data collection was adopted, which was considered suitable for the nature of the study. It employed a six-item online survey created by the author and revised by experts in the field, which was distributed to faculty members affiliated with university English departments in the five main Saudi regions selected by convenience sampling. The survey used a five-point scale (*always*, *often*, *sometimes*, *rarely*, and *never*). The six items were presented in English and measured how men and women used English in their departments to understand how they formulated and practised their own policies. The English departments are parts of Saudi universities offering academic programmes relevant to English studies (including English literature, linguistics, and education).

The participants were academics of various ranks, ranging from teaching assistants to full professors. Most participants obtained some of their degrees in international English-speaking countries (such as the US, Canada, UK, and Australia). A total of 210 valid responses were obtained (143 from women and 67 from men). The study’s purpose was explained in the introduction of the online survey, and participants were informed that participation was voluntary and that they could withdraw at any time. In addition, participants were assured of the confidentiality of their data.

## Results

Analysis of the six survey items was conducted using IBM SPSS Statistics, and responses from men and women were compared using the Mann-Whitney (MW) test to investigate whether there were statistically significant gender differences with regard to self-directed ELPs. This non-parametric test was chosen because the study compared differences between ordinal data obtained from two independent samples [60, 61]. The analysis of the six items mainly focused on the clustering of participants’ responses because the study predominantly aimed to focus on general differences between the two genders. Median values are provided to allow for a greater representation of the results.

In response to Item 1, most women (72.7%) reported that they ‘always’ used English for communication in committee meetings, while a smaller majority of men (52.2%) reported the same (Table 1). This indicates a 20.5% gender difference. Most other responses were also clear indicators that women have a higher tendency to use English in official meetings. For example, more men reported ‘rarely’ using English than women (10.4% vs. 2.1%). The median value of the responses for both men and women was 5.

**Table 1:** Use of English during committee meetings.

			Gender		Total
			Women	Men	
Item 1: *I use English for communication during committee meetings*	Always	Count	104	37	141
		%	72.7%	52.2%	67.2%
	Often	Count	24	13	37
		%	16.8%	19.4%	17.6%
	Sometimes	Count	9	7	16
		%	6.3%	10.4%	7.6%
	Rarely	Count	3	7	10
		%	2.1%	10.4%	4.8%
	Never	Count	3	3	6
		%	2.1%	4.5%	2.9%
Total		Count	143	67	210
		%	100.0%	100.0%	100.0%

The MW test was run on the responses to investigate the significance of the overall observed gender differences. The test results suggested that the difference was statistically significant at a *p-value* of 0.003, with an MW value of 3645.500 (Table 2). Therefore, in conclusion, most men and women in Saudi English departments tend to use English during official meetings, with significantly more women doing so.

**Table 2:** Mann-Whitney test.

Variable	Gender	N	Median	Mean Ranks	Sum of Ranks	Mann-Whitney	Z	Sig.
Item 1: *I use English for communication during committee meetings*	Men	67	5.00	89.08	5790.50	3645.500	-2.986	.003
	Women	143	5.00	111.51	15945.50			
	Total	210	-	-	-			

As for Item 2, most women (88.1%) reported that they ‘always’ used English for email correspondence with other faculty members; a smaller majority of men (67.2%) reported the same (Table 3). This reveals a 20.9% difference between the two groups. As with the findings from Item 1, most remaining responses also indicated that women have a greater tendency to use English in email correspondence. For instance, 9% of men reported ‘rarely’ using English in email correspondence, while only 1.4% of women reported the same. The median value of the responses for both men and women was 5.

**Table 3:** Use of English for email correspondence.

			Gender		Total
			Women	Men	
Item 2: *I use English for email correspondence with other members of the department*	Always	Count	126	45	171
		%	88.1%	67.2%	81.5%
	Often	Count	13	6	19
		%	9.1%	9.0%	9.0%
	Sometimes	Count	1	8	9
		%	0.7%	11.9%	4.3%
	Rarely	Count	2	6	8
		%	1.4%	9.0%	3.8%
	Never	Count	1	2	3
		%	0.7%	3.0%	1.4%
Total		Count	143	67	210
		%	100.0%	100.0%	100.0%

MW test results suggested that the difference was statistically significant at a *p-value* of 0.000, with an MW value of 3544.000 (Table 4). Therefore, in conclusion, most men and women in Saudi English departments use English for email correspondence, with a significantly larger majority among women.

**Table 4:** Mann-Whitney test.

Variable	Gender	N	Median	Mean Ranks	Sum of Ranks	Mann-Whitney	Z	Sig.
Item 2: *I use English for email correspondence with other members of the department*	Men	67	5.00	87.52	5689.00	3544.000	-4.032	.000
	Women	143	5.00	112.22	16047.00			
	Total	210	-	-	-			

Regarding using English for different types of formal communication, a large percentage of women (63.6%) reported ‘always’ using English for these purposes, while a smaller percentage of men (49.3%) reported the same (Table 5). The difference in responses between the two genders was 14.3%. The percentages of men and women who reported that they ‘often’ used English for these purposes were similar (men = 25.4%; women = 23.1%), with a difference of only 2.3%. The median value of the responses for both men and women was 5.

**Table 5:** English use for other types of formal communication.

			Gender		Total
			Women	Men	
Item 3: *I use English for other types of formal communication*, *such as announcements*, *signs*, *and postings*	Always	Count	91	33	124
		%	63.6%	49.3%	58.1%
	Often	Count	33	17	50
		%	23.1%	25.4%	23.8%
	Sometimes	Count	11	10	21
		%	7.7%	14.9%	10.0%
	Rarely	Count	5	5	10
		%	3.5%	7.5%	4.8%
	Never	Count	3	2	5
		%	2.1%	3.0%	2.4%
Total		Count	143	67	210
		%	100.0%	100.0%	100.0%

MW results suggested that the difference was statistically significant at a *p-value* of 0.016, with an MW value of 3791.500 (Table 6). Therefore, in conclusion, most men and women in Saudi English departments always or often use English in different types of formal communication, with a significant difference found in favour of women.

**Table 6:** Mann-Whitney test.

Variable	Gender	N	Median	Mean Ranks	Sum of Ranks	Mann-Whitney	Z	Sig.
Item 3: *I use English for other types of formal communication*, *such as announcements*, *signs*, *and postings*	Men	67	5.00	91.33	5936.50	3791.500	-2.404	.016
	Women	143	5.00	110.49	15799.50			
	Total	210	-	-	-			

Regarding Item 4, Table 7 shows that 71.3% of women ‘always’ communicate with students in English, while only 50.8% of men do the same. Moreover, only 20.3% of women ‘often’ used English for student communication compared to 40.3% of men. Hence, significant gender differences exist (20.5% difference in favour of women for ‘always’ and 20% in favour of men for ‘often’). The median value of the responses for men was 4 and that for women was 5.

**Table 7:** English use for communication with students.

			Gender		Total
			Women	Men	
Item 4: *I use English whenever I communicate with students*	Always	Count	102	34	136
		%	71.3%	50.8%	66.8%
	Often	Count	29	27	56
		%	20.3%	40.3%	26.7%
	Sometimes	Count	10	3	13
		%	7.0%	4.5%	6.2%
	Rarely	Count	2	3	5
		%	1.4%	4.5%	2.4%
Total		Count	143	67	210
		%	100.0%	100.0%	100.0%

MW test results suggested that the difference was statistically significant at a *p-value* of 0.005, with an MW value of 3683.500 (Table 8). Therefore, in conclusion, most men and women in Saudi English departments tend to use English when communicating with their students, with a significant difference found in favour of women.

**Table 8:** Mann-Whitney test.

Variable	Gender	N	Median	Mean Ranks	Sum of Ranks	Mann-Whitney	Z	Sig.
Item 4: *I use English whenever I communicate with students*	Men	67	4.00	89.67	5828.50	3683.500	-2.838	.005
	Women	143	5.00	111.24	15907.50			
	Total	210	-	-	-			

In response to Item 5, most women (55.2%) reported ‘always’ using English for various types of communication during work hours, while a smaller proportion of men (29.9%) reported the same (Table 9), representing a 25.3% difference. Further, the proportion of women who reported ‘often’ using English in such communications (32.9%) was smaller than men (38.8%). The median value in the responses for men is 4 and that for women is 5.

**Table 9:** English use for all types of formal and informal communication.

			Gender		Total
			Women	Men	
Item 5: *I use English for any sort of formal and informal communication during work hours*	Always	Count	79	20	99
		%	55.2%	29.9%	46.2%
	Often	Count	47	26	73
		%	32.9%	38.8%	34.8%
	Sometimes	Count	13	12	25
		%	9.1%	17.9%	11.9%
	Rarely	Count	4	8	12
		%	2.8%	11.9%	5.7%
	Never	Count	0	1	1
		%	0.0%	1.5%	0.5%
Total		Count	143	67	210
		%	100.0%	100.0%	100.0%

MW test results suggested that the difference was statistically significant at a *p-value* of 0.000, with an MW value of 3058.000 (Table 10). Therefore, in conclusion, most men and women in Saudi English departments always or often use English to communicate with one another, including both formal and informal situations. Moreover, a statistically significant difference in use was found in favour of women.

**Table 10:** Mann-Whitney test.

Variable	Gender	N	Median	Mean Ranks	Sum of Ranks	Mann-Whitney	Z	Sig.
Item 5: *I use English for any sort of formal and informal communication during work hours*	Men	67	4.00	80.05	5203.00	3058.000	-4.276	.000
	Women	143	5.00	115.62	16533.00			
	Total	210	-	-	-			

In a more specific enquiry (Item 6), 37.8% of women reported ‘always’ using English in their own private (non-academic) encounters with others, while a smaller proportion of men (25.4%) reported the same (Table 11). The difference between the two groups was 12.4%. Additionally, more women (35%) than men (23.9%) ‘often’ used English in these situations. Regarding infrequent use, a larger proportion of men (29.9%) reported occasional use of English in these situations compared to women (18.2%). The median value of the responses for men was 4 and that for women was 5.

**Table 11:** English use in private (non-academic) situations.

			Gender		Total
			Women	Men	
Item 6: *I use English in private (non-academic) situations with other members of the department*	Always	Count	54	17	71
		%	37.8%	25.4%	35.9%
	Often	Count	50	16	66
		%	35.0%	23.9%	31.4%
	Sometimes	Count	26	20	46
		%	18.2%	29.9%	21.9%
	Rarely	Count	8	10	18
		%	5.6%	14.9%	8.6%
	Never	Count	5	4	9
		%	3.5%	6.0%	4.3%
Total		Count	143	67	210
		%	100.0%	100.0%	100.0%

MW test results suggested that the difference was statistically significant at a *p-value* of 0.001, with an MW value of 3384.000 (Table 12). Therefore, in conclusion, most men and women in Saudi English departments tend to use English in informal private situations, with a significant difference between the two in favour of women.

**Table 12:** Mann-Whitney test.

Variable	Gender	N	Median	Mean Ranks	Sum of Ranks	Mann-Whitney	Z	Sig.
Item 6: *I use English in private (non-academic) situations with other members of the department*	Men	67	4.00	85.06	5529.00	3384.000	-3.274	.001
	Women	143	5.00	113.34	16207.00			
	Total	210	-	-	-			

## Discussion and conclusion

The Saudi higher education system offers English language programmes in an EFL context through various English language departments. In these departments, academics from both genders are employed from Saudi Arabia as well as other regional and international countries. Members in these departments interact with one another, for both official work-related and non-official private purposes. The literature suggests that these departments do not employ formal ELPs governing how languages should be used [5]. This has precipitated the emergence of self-directed policies, which are freely practised by faculty members. This disorganisation may undermine an institution’s professional image in the eyes of its students, other beneficiaries, and stakeholders; the attractiveness of the institutional environment may also be decreased. Moreover, it may become a starting point for internal conflict [20, 31] between faculty members themselves, where some may use Arabic (L1) in the presence of non-Arabic speaking staff, which is considered impolite and provocative. As such, ELPs in this context are important to relevant policymakers. The literature suggests that the genesis of such policies can be bottom-up (micro) or top-down (macro) [16, 17]. This study investigated gender differences in the self-directed policies practised by faculty members in Saudi English departments. Arguably, these policies form the basis of micro-level ELPs and, thus, are important to policymakers.

The findings of the study show that most academics of both genders in these departments use English to communicate in formal and informal situations at the department level. Differences in language use between the two genders were found to exist in all six survey items; these were statistically significant for all items. The results show that women show greater commitment to English language use than men. This may suggest that women are likely to opt for stricter ELPs (i.e., they would favour policies that promote more English language use). The commitment of women to using English in non-official situations—such as talking with a colleague about an incident that occurred at a social event—is indicative of how attached they are to the language and high status that they accord it. Although men reported using English in similar situations, they did not show the same level of commitment. Speculatively, such differences might mean that men and women will not agree in decision-making concerning ELPs.

### Pedagogical implications

An important factor in the success of an ELP imposed by policymakers is its acceptance by the community that it affects. The significant differences identified here in terms of the policies practised by each gender suggest that they will have different views on the framework of any future ELPs. This creates a challenging situation for policymakers, wherein it may not be easily feasible to introduce macro policies that are accepted and successfully practised by all faculty members. Consequently, encouraging the development of these policies at the micro-level is advisable, starting from the members of these departments. Additionally, since differences exist, the individuals responsible for shaping policies should involve *both* genders to allow for mediation between their different approaches, thus resulting in more widely accepted joint decisions.

The fact that most faculty members seemingly use English in formal and informal situations entails a few ramifications. First, these faculty are likely to believe that English should be used more often because they work in an English department and all members speak the language; therefore, using English at the department level is a professional requirement, especially when non-Arabic speaking faculty are present. Second, the Saudi EFL context is known for its lack of opportunities to practise professional academic English, and, as a result, the department is one of the few venues for professional language practice, which helps develop and maintain linguistic proficiency.

Overall, the findings of the study offer insights for policymakers concerned with the overall framework of ELPs that can be accepted by faculty members. In general, policies are expected to limit L1 and encourage the frequent use of English. Policymakers may also accept English-only policies. If such policies were to be introduced, the academic services offered by these departments could be more easily internationalised [21, 36]. Additionally, these departments will be among the first Saudi governmental education institutions to create an environment attractive to international students and other professionals.

The current study revealed that gender differences exist among faculty members in Saudi higher education institutions. Such differences must be addressed carefully to avoid any possible conflicts and achieve effective best practices. In general, educational policies require a thorough and well-thought-out design before being implemented, as well as the involvement of representative members of the concerned communities. Such involvement will increase the likelihood that policies are accepted and followed, and achieve the purposes for which they were introduced. In terms of limitations, this study did not reveal the specific details of the self-directed policies the two genders practise in their daily work, which forms an area for future research. Furthermore, investigating the relationship between ELPs both inside and outside the classroom would be interesting. ELPs in the Saudi context have proven to be a promising area for research; further research is required to situate English usage most effectively in this context.

## References

[1] Coleman H, Capstick T. Language in education in Pakistan: Recommendations for policy and practice. Islamabad: British Council; 2012.

[2] Tikly L. Education and the new imperialism. Comp Educ. 2004;40: 173–198. doi: 10.1080/0305006042000231347

[3] Sperduti V. Internationalization as westernization in higher education. Comp Int Educ. 2017;9: 9–12.

[4] Alnasser SMN. Gender differences in beliefs about English language policies (ELPs): The case of Saudi higher education English departments. Int J Educ Literacy Stud. 2018a;6: 111–118. doi: 10.7575/aiac.ijels.v.6n.2p.111

[5] Almoaily M, Alnasser SMN. Current English language policies in Saudi Arabian higher education English departments: A study Beyond the domain of the classroom. J Arts. 2019;31: 35–47.

[6] Al-Issa A, Dahan L. Global English and Arabic: Issues of language culture and identity. Oxford: Peter Lang Publishing; 2011.

[7] Khalaf S. Globalization and heritage revival in the Gulf: An anthropological look at Dubai heritage village. Soc Aff. 2002;19: 13–42.

[8] Khalaf S. National dress and the construction of emirati cultural identity. J Hum Sci. Bahrain: University of Bahrain. 2005.

[9] Payne M, Almansour M. Foreign language planning in Saudi Arabia: Beyond English. Curr Issues Lang Plan. 2014;15: 327–342. doi: 10.1080/14664208.2014.915461

[10] Deumert A. Language planning and policy. In: Meshtrie R, Swann J, Deumert A, Leap WL, editors. Introducing sociolinguistics. Edinburgh: Edinburgh University Press; 2005. pp. 384–418.

[11] Fishman J. Advances in language planning. The Hague: Mouton; 1974.

[12] Karam F. Toward a definition of language planning. In: Fishman JA, editor, Advances in language planning. The Hague: Mouton; 1974. pp. 103–124.

[13] Jernudd B, Gupta J. Towards a theory of language planning. In: Rubin J, Jernudd B, editors, Can language be planned? Sociolinguistic theory and practice for developing nations. Hawaii: The University Press of Hawaii; 1971. pp. 195–215.

[14] Tauli V. The theory of language planning. In: Fishman JA, editor. Advances in language planning. The Hague: Mouton; 1974. pp. 49–67.

[15] Kaplan RB, Baldauf RB. Language planning: From practice to theory. Clevedon: Multilingual Matters Ltd; 1997.

[16] Johnson DC. Language policy. Hampshire. UK: MacMillan; 2013.

[17] Schiffman HF. Linguistic culture and language policy. London: Routledge; 1996.

[18] Badry F, Willoughby J. Higher education revolutions: Short-term success Versus long-term viability? In: Badry F, Willoughby J, editors Higher education revolutions in the Gulf. New York: Routledge; 2016. pp. 204–236.

[19] Alhendi O. Language policy and economics: Does English language accelerate the wheel of development in the economies or not? A review. The annals of the University of Oradea. Econ Sci. 2019;2: 366–379.

[20] Tickoo ML. Language in education. World Englishes. 2006;25: 167–176. doi: 10.1111/j.0083-2919.2006.00454.x

[21] Conceição MC. Language policies and internationalization of higher education. Eur J Higher Educ. 2020;10: 231–240. doi: 10.1080/21568235.2020.1778500

[22] Wachter B, Maiworm F 2008. English. Taught programmes in European higher education. The picture in 2007. ACA papers on cooperation in education. Bonn: Lemmens.

[23] Coleman JA. English-Medium teaching in European higher education. Lang Teach. 2006;39: 1–14. doi: 10.1017/S026144480600320X

[24] Coleman H. Teaching and learning in Pakistan: The role of language in education. Islamabad: The British Council; 2010.

[25] Kachru BB. The alchemy of English and the spread, functions and models of non-native englishes. Oxford: Pergamon Press; 1986.

[26] Widdowson HG. Routledge encyclopedia of language teaching and learning. London: Routledge; 2000 English in Michael Byram (Ed.). pp. 193–196.

[27] Gunnarsson BL. Swedish, English. French or German—The language situation at Swedish universities. In: Ammon U, editor, The dominance of English as a language of science. Effects on other languages and language communities. Berlin, New York: Mouton de Gruyter; 2001. pp. 229–316.

[28] Kuteeva M, Airey J. Disciplinary differences in the use of English in higher education: Reflections on recent language policy developments. High Educ. 2014;67: 533–549. doi: 10.1007/s10734-013-9660-6

[29] Benson C. The importance of mother tongue-based schooling for educational quality. Background paper prepared for the ‘Education for All’ Global Monitoring Report 2005; 2004. The Quality Imperative. Available from: http://unesdoc.unesco.org/images/0014/001466/146632e.pdf. United Nations Educational, Scientific and Cultural Organization.

[30] Trudell B. Local-language literacy and sustainable development in Africa. Int J Educ Dev. 2009;29: 73–79. doi: 10.1016/j.ijedudev.2008.07.002

[31] Adriano M, Framco N, Estrella E. Language-in-education policies and stakeholders’ perception of the current MTB-MLE policy in an ASEAN country. Aust J Lang Lit. 2021;44: 84–100.

[32] Liddicoat AJ, Kirkpatrick A. Dimensions of language education policy in Asia. J Asian Pac Commun. 2020;30: 7–33. doi: 10.1075/japc.00043.kir

[33] Eno MA, Ahad AM, Shafat A. Perceptions of academic administrators and policymakers on ESL/EFL education. J Somali Stud. 2019;6: 93–119. doi: 10.31920/2056-5682/2019/V6n1a4

[34] Ek AC, Ideland M, Jönsson S, Malmberg C. The tension Between marketisation and academisation in higher education. Stud Higher Educ. 2013;38: 1305–1318. doi: 10.1080/03075079.2011.619656

[35] Airey J, Linder C. Bilingual scientific literacy? The use of English in Swedish university science programmes. Nord J Engl Stud. 2008;7: 145–161. doi: 10.35360/njes.105

[36] Guo Y, Guo S, Yochim L, Liu X. Internationalization of Chinese higher education: Is it Westernalisation? J Stud Int Educ. 2021: 1–18.

[37] Bolton K, Kuteeva M. English as an academic language at a Swedish university: Parallel language use and the ‘threat’ of English. J Multiling Multicult Dev. 2012;33: 429–447.

[38] Bartlett L, García O. Additive schooling in subtractive times: Bilingual education and Dominican immigration youth in the heights. Nashville: Vanderbilt University Press; 2011.

[39] García O, Kleifgen JA, Falchi L. From English language learners to emergent bilinguals. Equity Matters Res Rev. 2008;1: 1–61.

[40] Hamm-Rodriguez M, Morales AS. (Re)producing insecurity for Puerto Rican students in Florida Schools: A Raciolinguistic perspective on English-only policies. Cen J. 2021;33: 112–132.

[41] Valenzuela A. Subtractive schooling: U.S.-Mexican youth and the politics of caring. New York: State University of New York Press; 1999.

[42] Jenkins J, Wingate U. Staff and student perceptions of English language policies and practices in ‘international’ universities: A UK case study. Higher Educ Rev. 2015;47: 47–73.

[43] Menken K. English learners left Behind: Standardized testing as language policy. Bristol, UK: Multilingual Matters; 2008.

[44] Turner J. Language in the academy. Cultural reflexivity and intercultural dynamics. Bristol: Multilingual Matters; 2011.

[45] Fishman JA. Holy languages in the context of societal bilingualism. Contribs Sociol Lang. 2002;87: 15–24.

[46] Liddicoat AJ. Language planning as an element of religious practice. Curr Issues Lang Plan. 2012;13: 121–144. doi: 10.1080/14664208.2012.686437

[47] Alnasser SMN. Investigating English language policies in Saudi higher education English departments: Staff members’ beliefs. Linguist Lit Stud. 2018;6: 157–168. doi: 10.13189/lls.2018.060402

[48] Elyas T, Badawood O. English language educational policy in. Saudi Arabia Post 21st Century: Enacted Curriculum, Identity, and Modernisation: A Critical Discourse Analysis Approach. Fire; 2016, 3: 70–81.

[49] Bacon SMC, Finnemann MD. Sex differences in self-reported beliefs about foreign language learning and authentic oral and written input. Lang Learn. 1992;42: 471–495. doi: 10.1111/j.1467-1770.1992.tb01041.x

[50] Javid CZ. A gender-based investigation of the perception of English language teachers at Saudi universities regarding the factors influencing learner autonomy. Arb Wor Eng J. 2018;9: 310–323.

[51] Oxford RL. Use of language learning strategies: A synthesis of studies with implications for strategy training. System. 1989;17: 235–247. doi: 10.1016/0346-251X(89)90036-5

[52] Alhaysony M. Language learning strategies use by Saudi EFL students: The effect of duration of English language study and gender. Theo Prac Lang Stud. 2017; 7: 18–28.

[53] Calafato R, Tang F. Multilingualism and gender in the UAE: A look at the motivational selves of Emirati teenagers. System. 2019;84: 133–144.

[54] Yılmaz C. The relationship between language learning strategies, gender, proficiency and self-efficacy beliefs: A study of ELT learners in turkey. Procedia Soc Behav Sci. 2010;2: 682–687. doi: 10.1016/j.sbspro.2010.03.084

[55] Tannen D. Gender and discourse. Oxford: Oxford University Press; 1995.

[56] Herring SC. Gender differences in CMC: Findings and implications. Comput Prof Soc Respons J. 2000;18.

[57] Leaper C, Ayres MM. A meta-analytic review of gender variations in adults’ language use: Talkativeness, affiliative speech, and assertive speech. Pers Soc Psychol Rev. 2007;11: 328–363. doi: 10.1177/1088868307302221 18453467

[58] Bernat E, Lloyd R. Exploring the gender effect on EFL learners’ beliefs about language learning. Aust J Educ Dev Psychol. 2007;7: 79–91.

[59] Widodo H, Elyas T. Introduction to gender in language education. Sexu Cultu. 2020;24: 1019–1027.

[60] Gray C D, Kinnear P R. IBM SPSS statistics 19 made simple. London, UK: Psychology Press; 2012.

[61] Larsen-Hall J. A guide to doing statistics in second language research using SPSS. New York, NY: Routledge; 2010.

